# Supplementary material for: Chromothripsis during telomere crisis is independent of NHEJ, and consistent with a replicative origin
Source: Genome Res. 2019 May;29(5):737–49. doi: 10.1101/gr.240705.118 (PMC6499312; doi:10.1101/gr.240705.118)
Supplement: Supplemental Material [file supp_gr.240705.118_Supplemental_file_1.zip › contigs/annotated_contigs/DB56/contig.3.DB56_length_468_mean_cov_9.17521367521.docx]

**DB56_length_468_mean_cov_9.17521367521**

```
GGAACTAGAATTCAAGATGAGATTTGGGTGGGGACAAAGTCAAACCATATCTTTACATATGTGTCTATGTGTGTGTCCAGTTTTTACTA
 >chr13:92233397-92233617 + E=7e-121 p=0e+00
TGTAGTATATACTATATTCTGTGTACTCTAAAAAGTGTAGAGACTTTTAGAGAAAATAAAAAGTAGATAGGAAAGTAGAGTGGAAGAAA

TATAACACTATTTTGGCCCCATCATGGTCAGCTTTTGTAAAG|CTGTTTGTATATATATAT|AGAGAGAGAGAGAGAGAGAGAGGGAGA
                                                             >chr13:92238823-92239004 +
GGAAGTGCTACATTTTTAAACCATCAGATCTCATGAGAACTCACTATCATGAGAACATCATGGGGGGACTGCCCCCATGATCCAATCAC
 E=2e-97 p=0e+00
CCCCCAGCAGGTCCCTTTCTTGATATGTGGGGATTAT|AATTCAAGATGAGATTTGGGTGGGGACA|AAGTCAAACCATATCTTTACAT
                                      >chr13:92233405-92233482 + E=9e-35
ATGTGTCTATGTGTGTGTCCAGTTTTT
```
